# A Composite Fabric-Based Soft Rehabilitation Glove With Soft Joint for Dementia in Parkinson’s Disease

**DOI:** 10.1109/JTEHM.2020.2981926

**Published:** 2020-03-19

**Authors:** Yinglong Chen, Xinyan Tan, Di Yan, Zengmeng Zhang, Yongjun Gong

**Affiliations:** Naval Architecture and Ocean Engineering CollegeDalian Maritime University 12421 Dalian116026China

**Keywords:** Soft robotics, soft rehabilitation glove, wearable exoskeleton, dementia in Parkinson’s disease

## Abstract

A kind of wearable exoskeleton soft rehabilitation glove is proposed for the dementia in Parkinson’s disease (PD) patients with loss of hand function, limited range of motion, and insufficient finger muscle strength to carry out rehabilitation exercise training in a passive or auxiliary way. A novel soft joint structure based on composite fabric material is introduced for the design of the soft glove with bionic method, and experiments are conducted to verify the effeteness of the proposed soft rehabilitation glove. The test results showed that when the fluid pressure was 0.42 MPa, the joint angle of MCP, PIP and DIP could be up to 81°, 98°, 72°, and produce output torque of 1.18Nm, 1.44Nm and 1.82Nm respectively, which meets the requirements of the hand rehabilitation. A dynamic rehabilitation-training test of the rehabilitation glove was also carried out, and the results showed that the movement frequency of soft fingers could reach 30 times/min, which is sufficient for repetitive flexion/extension exercise. In order to verify the grasping characteristics of the soft glove for irregular objects, experiments were carried out. The experimental results showed that the bionic soft glove was dexterous in grasping, which conforms to the universal grasping characteristics of human hands, has the function of assisting daily life (ADL), and meets the requirements of rehabilitation.

## Graphical abstract

A novel wearable exoskeleton soft rehabilitation glove with soft joint is proposed for the dementia in Parkinson's disease (PD), and continuous passive training test is conducted through sEMG and fluid-driven proportional control system.

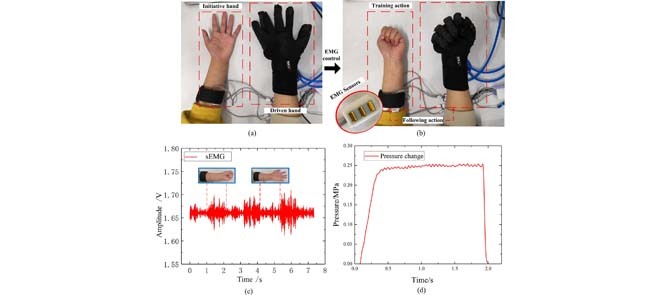

## I. Introduction

Dementia represents arguably the most significant social, economic, and medical crisis of our time, which is a syndrome of severe cognitive loss caused by illness or injury. Dementia caused by traumatic brain injury is usually static, while dementia caused by neurodegenerative diseases such as Alzheimer’s disease is usually progressive and can eventually be fatal. Age, neuropsychological damage, and the severity of Parkinson’s disease (PD) are risk factors for dementia, and late-onset PD is associated with a higher risk of dementia [1]. PD is one of the most common progressive neurodegenerative disorders, leading to loss of motor function and decreased quality of life. A study showed that exercise can trigger several plasticity-related events in the brain of PD patients, including cortical motor excitement, increasing and decreasing gray matter volume, and altering BDNF levels [2]. Increasing evidence supports the role of exercise in improving both motor and non-motor outcomes in PD [3], [4]. Studies on normal aging have shown that there may be a variety of potential effects of increased exercise capacity, including improved limb strength and flexibility, proprioceptive and postural awareness, neuromuscular coordination and reaction time, performance, and reduced fear of falling [5]. According to the survey, 48.7% of quadriplegic patients indicated that the recovery of arm and hand functions would significantly help improve their quality of life [6]. At the earliest stage, this kind of patients’ hand rehabilitation training mainly adopts the one-to-one adjuvant treatment by therapists [7], [8]. Rehabilitation of the hands with a therapist can help restore basic motor function, however intensive physical therapy usually involves the use of repetitive task exercise (RTP), which is inefficient, time-consuming and laborious. Therefore, the rehabilitation needs of such patients will be a huge challenge to the technology.

Robotics is being proposed as a solution to assist hand function recovery, and is being used to assist patients with nerve damage in hand rehabilitation training and a variety of grasping functions through a wearable driving platform, the robotic exoskeleton system. Traditional hand exoskeleton is composed of dc motor [9], [10], linear actuator [11], [12], rigid connecting rod and other rigid parts [13], [14], and their stiffness, weight and constraints on joints freedom cause complications [15], [16]. Compared with artificial physiotherapist, rehabilitation robots have many potential advantages: It can provide sustained, accurate and quantitative motor stimulation for the affected limb, the training process is tested and feedback to facilitate system evaluation, multiple training modes, and it is easy to make rehabilitation equipment portable, family-oriented and intelligent, benefiting more patients [17], [18].

As a new product in the field of robotics, the emergence of soft robots brings a new dawn to the hand rehabilitation of such patients. Compared with the traditional rigid hand rehabilitation robots, this kind of robot not only has many advantages of the traditional robots, but has the advantages of lighter in weight, higher in safety coefficient, more convenient and more versatile, and requires only simple structural design to achieve a higher degree of freedom. Its soft actuators are mainly driven by cable [19], [20], fluid [21], [22], and smart material deformation [23], [24], which combine the finger structure design based on fabric, and without complicated mechanical settings. Such an exoskeleton can reduce the possibility of misaligned compression and shear forces on joints [25]–[29].

Combining based on the fabric gloves with inflatable soft actuators, several research groups have developed wearable exoskeleton soft robotics of hand. For example, Polygerinos *et al*. have developed an assisted rehabilitation soft robot [30]. The molded elastomer chamber and the fiber-reinforced parts constitute the soft actuators, and the fiber-reinforced parts cause specific bending, distortion and extension trajectories under fluid pressure. These soft actuators are designed through mechanical structure to match and support the range of motion of each finger. Cappello *et al*. [31] have developed a pneumatic rehabilitation glove based on fabric by using the anisotropy of textile. By combining the fabric layers of two different materials, an air bag made of TPE thermoplastic elastomer material is wrapped inside, when the soft unit is pressed, it can generate bending movement. Based on the novel 3D printing technology, Ang and Yeow [32] have developed a set of fully 3D printed exoskeleton hand rehabilitation assistant soft robotic system. The printing material is made of thermoplastic elastomer, which can be washed and reused with almost no maintenance and low production cost. Hong *et al.* [33]–[35], have developed a soft rehabilitation glove with embedded corrugated fabric layer. The soft glove is made of silica gel material with internal channel and a corrugated mechanical structure on the upper surface. Its soft joints are made by alternating lamination of extendable and non-extendable fabrics on the lower surface, and pleated fabric layers are laminated on the corrugated structure of the upper surface to prevent excessive expansion of silica gel. Based on this research, the group then developed a full-fabric soft rehabilitation glove. Its fabric layer material is selected as polyester knitwear, and designed as a mechanical structure with pleats, and the interior is covered with airbags of latex material. Compared with the semi-fabric soft gloves developed in the first generation, this kind of gloves can achieve a larger terminal output force with a lower driving pressure.

As introduced above, the soft robotics for hand rehabilitation are lighter, smaller in shape and cheaper to make comparing with rigid gloves. Through the combination of fabric layers with different materials (the fabric layer on the top was designed with wrinkles in the warp direction, and the bottom layer was designed the non-stretchable fabric layer), the bending movement can be generated through the differential torque of the layers. However, the finger phalanges of the soft rehabilitation device usually presented a circular arc shape, and the motion trajectory of the joint during rehabilitation was not consistent with that of the patient’s finger. In addition, the output force of the soft finger is limited due to the selection and configuration of reinforcing fibers or fabrics. For example, the density of reinforcing fibers decreases with the increase of radial length, which greatly limits the driving pressure and reduces the driving moment of the fluid actuator.

In this work, combining the new application of soft robot technology and the 3D printing technology, a novel wearable exoskeleton soft rehabilitation glove with soft joint structure based on composite fabric material is proposed. The soft joint is innovatively designed to make the movement mechanism of soft gloves more consistent with the human hands based on study the fabric-reinforced joint soft rehabilitation glove. The fabric adopted in this paper is a typical anisotropic knitting material with good axial elasticity and high radial modulus, which enable soft fingers supply greater output force. The device can be adopted by PD patients to carry out rehabilitation exercise training in passive or auxiliary way, so that PD patients can actively participate in it, and it is possible to delay and improve the progress of the disease, which has a positive effect on patients with dementia in PD.

## II. Structural Design

First, the structure and movement principle of the human hand is deeply studied. Based on the principle of bionics, the articular soft rehabilitation glove with soft joint imitating the structure and principle of human hands is designed to maximize the imitation of human hands’ function by using soft materials such as silica gel and composite elastic fabric. The 3D printed silica gel matrix is used as the inner layer and the composite elastic knitted fabric is wrapped externally as the reinforcing layer of the matrix. Then, the fabric layers are cut and stitched together to form a composite elastic fabric with unique structure, which adds joint structure to fingers of rehabilitation glove, and makes the soft finger structure same as the human hand structure, which has the phalange interphalangeal joints part.

### A. Design Requirements

Human fingers are composed of metacarpal, phalangeal and interphalangeal joints. According to the human anatomy, the human hand consists of five metacarpal bones and the corresponding phalanx of each metacarpal bone, and these bones are joined by many joints with little friction, has 21 DOFs, in which the thumb has 5 DOFs and the other four fingers have 4 DOFs respectively. It causes the track curvature of fingers to be variable when bending, which is called piecewise circular arc with variable curvature. At the same time, during finger grasping, only the interphalangeal joints can be bent because the phalanx part cannot be bent. In addition to the thumb, each finger is composed of distal phalanx (DP), middle phalanx (MP), proximal phalanx (PP) and distal interphalangeal joint (DIP), proximal interphalangeal joint (PIP), metacarpophalangeal joint (MCP) [36].

In human hand kinematics, each joint and finger are closely related and coupled with each other, mainly including two types: One is that the physical structure of the fingers constrains the range of motion of the joints. For example, while the distal interphalangeal joints are bent, the proximal interphalangeal joints are also slightly bent. The other kind of constraint is the movement between the joints and fingers. For example, except the thumb, the other four fingers also influence each other when moving [37]. According to the data, although the range of motion of the hand joints varies with each individual, there is generally a general range [38].

The hand has multiple redundant DOFs, and it can simplify the structure and facilitate the modular design under the premise of meeting the requirements of clinical rehabilitation sports training. In this work, the design parameters of rehabilitation finger and phalangeal bone were determined according to the size of the patient’s hand bone. The metacarpal bone was mainly used for the connection and fixation between soft fingers, and the design parameters only needed to meet the conditions of fixation and assembly. Design parameters of finger and metacarpal bones of soft rehabilitation gloves can be seen in Table 1. In addition, for patients with loss of hand function caused by stroke and other diseases, the maximum extension force perpendicular to the fingertip varies with the severity of the disease. If the auxiliary equipment is used to drive the finger movement, the maximum strength should be equal to the ideal value of 10 N.

**TABLE 1:** Design Parameters of Soft Finger Phalanx and Metacarpal Bone

Name	Thumb	Index finger	Middle finger	Ring finger	Little finger
Proximal phalanx (mm)	25	30	35	30	25
Middle phalanx (mm)	none	25	25	25	20
Distal phalanx (mm)	20	20	20	20	20
Metacarpale (mm)	15	15	15	15	15

### B. Overall Design of Soft Finger

The human hand is mainly composed of bones, joints and soft tissues (including skin, muscles, nerves, blood vessels, etc.). The hand rotates the joint during movement, and if the exoskeleton glove cannot follow this movement, it will create a shear force that causes pain in the joint [39]. Therefore, the natural range of motion (ROM) of the hand must be as close to the target as possible to allow for extensive functional motion. Therefore, the rehabilitation glove requires the soft element to have good bending characteristics, driving ability and minimum impedance.

**FIGURE 1.**
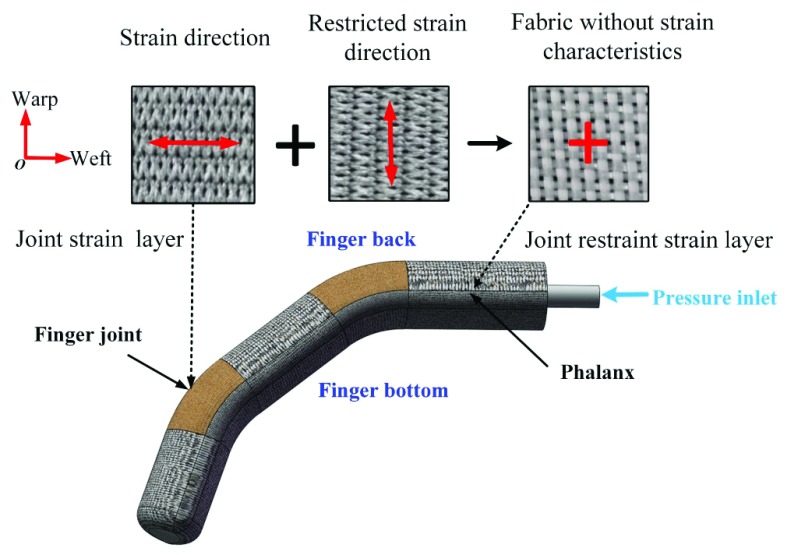
Schematic diagram of the composition of the joint fabric reinforced soft body rehabilitation finger.

In order to imitate the biological structure of human hand, we design the soft finger as the combination of phalanges and soft joints as shown in Figure 1. The phalanges will not deform, but the soft joints can approximate the movement of human finger joints. The layer of fabric at soft joints is composed of single knitted fabric arranged along the axial direction of silicone liner. Due to the unidirectional stretchability of knitted fabric, the joints can be elongated in the latitudinal direction while the meridional shape variable remains unchanged. The layer of fabric at the phalanx is composed of two layers of knitted fabric interlaced and combined. According to the warp and weft deformation characteristics of fabric materials, when two layers of fabric are combined together, the warp direction of the lower layer limits the weft deformation of the upper layer, similarly the warp direction of the upper layer also limits the weft deformation of the lower layer. This composite fabric layer is equivalent to the woven fabric layer. The result is that the metacarpal and phalangeal portions of the soft finger are neither elongated nor expanded in the latitudinal direction. In this way, the joint movement of soft fingers can be realized.

To sum up: through the different shape variables of the fabric layer material of the bottom and back of the finger, the soft finger can realize the joint bending action. The joint parts can produce flexible bending movement, and the metacarpal and phalanx parts always maintain the original shape during the joint bending process.

### C. Elastic Knitted Fabric

The soft joint requires that the elastic fabric has good axial elasticity and can limit the radial deformation. In this paper, one type of elastic knitted fabric is adopted which has anisotropy under stress due to the characteristics of warp and weft thread materials, the warp yarns are made of latex, and the weft yarns are polyester low-elastic yarns [40]. That is, the warp deformation is negligible and the weft deformation is significant when the knitted fabric is stressed. The enlarged physical picture of plain fabric can be seen in Figure 1. It is a composite warp-knitted fabric, through the combination of different yarns to form a fabric layer, it has high elastic modulus, low weight, better durability and stability, and it has excellent draping and seaming. At the same time, this kind of elastic knitted fabric is easy to purchase from the market at a relatively low price.

**FIGURE 2.**
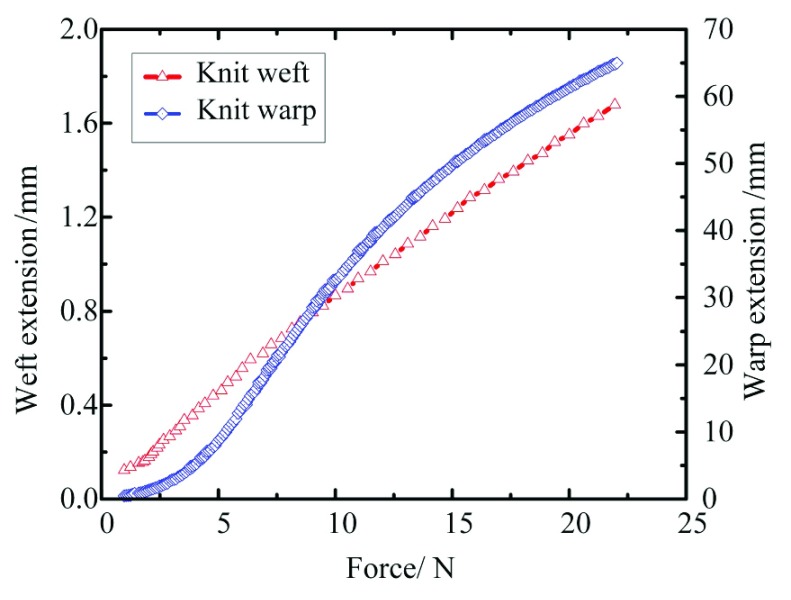
Mechanical properties of warp and weft tensile strain surface of the fabric.

In order to investigate the mechanical characteristics of the adopted elastic knitted fabric, the warp and weft tensile tests were carried out on the tensile testing machine. Experimental results report that under the condition that the maximum tension is 22N, the warp deformation is 1.76mm, the elongation is 2.7%, however when the weft deformation is 65mm, the elongation is 100%, and the weft deformation is 36.93 times of the warp deformation as shown in Figure 2. It proves that the elastic knitted fabric has well unidirectional stretchability and meets the design requirements of the soft joint.

### D. Silicon Matrix Structure

**FIGURE 3.**
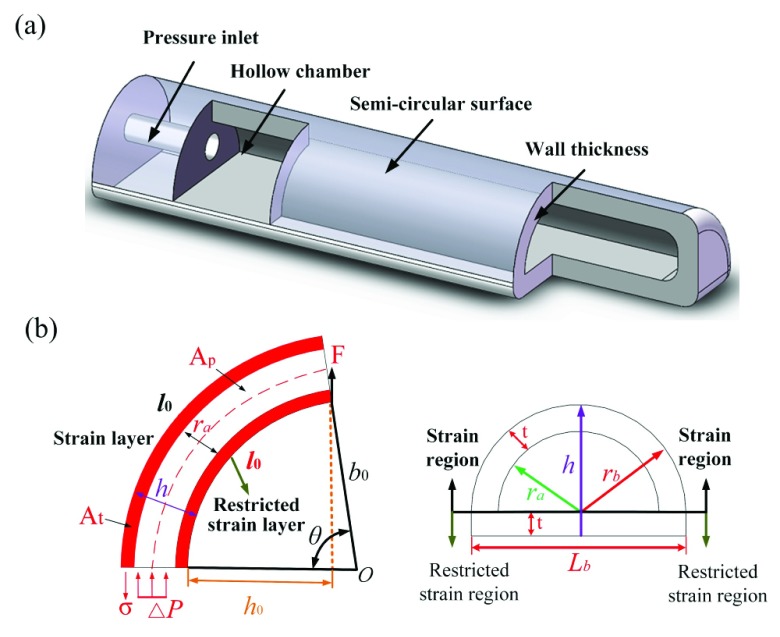
(a) The 3D model of the silica gel matrix. (b) The schematic diagram of soft finger joint stress distribution and cross section.

According to the contour characteristics of human hand, five fingers of soft rehabilitation glove have the similar silica gel matrix structure but differential parameters. The 3D model of the silica gel matrix is rendered in Figure 3(a). The outer contour of the silicone matrix is designed as a semicircle, and the inner of the matrix is a hollow structure, which is used to fill the driving fluid medium. One end of the matrix is enclosed and the other end is provided with a cylindrical pressure inlet.

It is assumed that the radial expansion of silica gel matrix and the change of wall thickness are not considered in the process of soft fingers rehabilitation, and soft joints are regarded as arcs with constant curvature. According to its bending action mechanism, in the process of soft joint bending, the length of the restricted strain layer at the joint remains unchanged, while the length of the strain layer increases gradually. The distribution of the stress state of the soft rehabilitation finger joint when it is under stress bending is rendered in Figure 3(b).

Due to the limitation of the precision of the 3D printer when preparing the mold, the success rate of the finished product preparation can only be improved by ensuring a certain wall thickness in the design of the silicon matrix structure. When the wall thickness of silicon matrix $t$ is 2 mm, the quality of the finished silicon matrix is higher. According to the above soft rehabilitation glove design requirements, the main design parameters of soft finger silicone matrix are shown in Table 2.

**TABLE 2:** Main Design Parameters of Silica Gel Matrix

Parameters(mm)	Values
The semicircular surface radius of the inner cavity of Silica gel matrix: $r_{\mathrm {a}}$	5.5
The surface radius of outer semicircle of silica gel Matrix: $r_{\mathrm {b}}$	7.5
Silica gel matrix wall thickness: $t$	2
Radial width of silica gel matrix: $h$	9.5

### E. Design of Soft Joint

**FIGURE 4.**
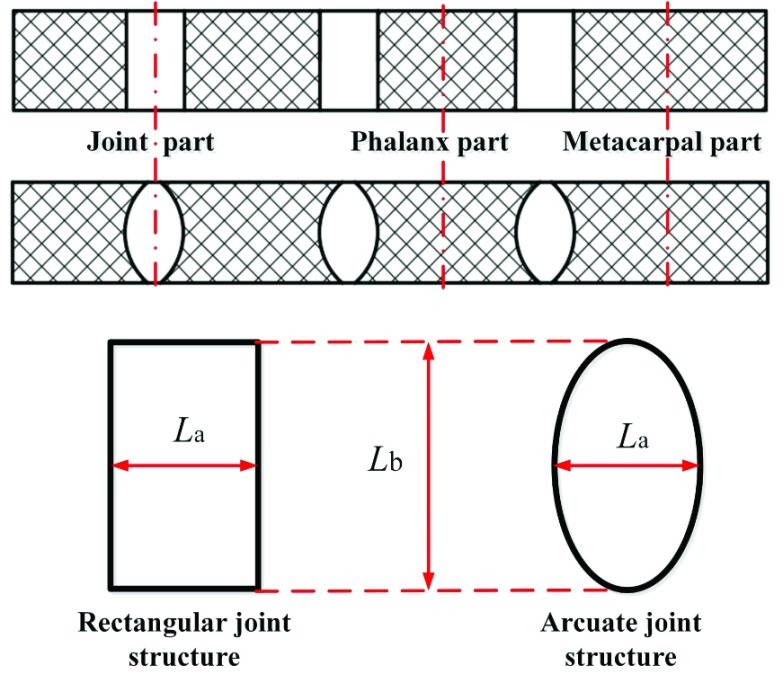
The fabric layer structure schematic diagram of soft joint.

In order to obtain good bending performance of imitating human hands for the soft finger, this work analyzes two types of soft joint structures: rectangular structure and elliptic structure as seen in Figure 4. The rectangular structure and elliptic structure refer to that the fabric material of the limit strain layer is cut into rectangular shape and elliptic shape respectively, and the zonal spacing of the two different structures was set as $L_{a}$ and the longitudinal width as $L_{b}$.

For the rectangular structure, the unstretched length of the strain layer fabric in the direction of the central axis is equal everywhere and equal to $L_{a}$. For the elliptic structure, the unstretched length of the strain layer fabric in the direction of the central axis is larger when it is close to the central axis of the curved structure, and the maximum value is $L_{a}$. The length becomes shorter when it is far away from the central axis. When it is at the two endpoints of the curved structure far from the central axis, the unstretched length of the strain layer fabric can be regarded as zero.

As shown in Figure 3(a), the bending angle $\theta $ for the soft joint can be given by

\begin{equation*} \theta =\frac {180\cdot (l_{n} -l_{0})}{\pi \cdot h}\tag{1}\end{equation*}

where $l_{0}$.is the length of the restricted strain layer, $l_{n}$ is the maximum length of the strain layer fabric of the joint ($L_{a}$ is the maximum unstretched length of the strain layer). We can then obtain the relationship between the bending angles of rectangular and elliptic structure by

\begin{equation*} \frac {\theta _{T}}{\theta _{J}}=\frac {l_{n}}{l_{n} -l_{0}}>1\tag{2}\end{equation*}

where $\theta _{J}$ is the bending angle for rectangular structure, and $\theta _{T}$ is the soft finger bending angle for elliptic structure with $l_{0}\approx 0$. As seen in Equation (2), the elliptic structure of the soft joint can produce larger bending angle than the rectangular structure of the soft joint with same fluid pressure. Therefore, the elliptic structure is more reasonable and it is adopted in this paper. In this paper, the unstretched length $L_{a}$ for MCP, PIP, DIP is set to 18 mm, 15 mm and 12 mm, respectively.

### F. Fabrication of Soft Glove

Silica gel matrix was prepared by casting liquid silica gel in the mold. Based on the structure and design parameters of silica gel matrix obtained by previous design, the design of casting mold was carried out. The mold is made by 3D printing, and the consumable material is PLA. This silica gel matrix preparation includes the following steps: (a) reagent modulation, (b) reagent degassing, (c) mold design, printing and assembly, (d) silica gel casting, (e) baking and curing, and (f) stripping molding.

**FIGURE 5.**
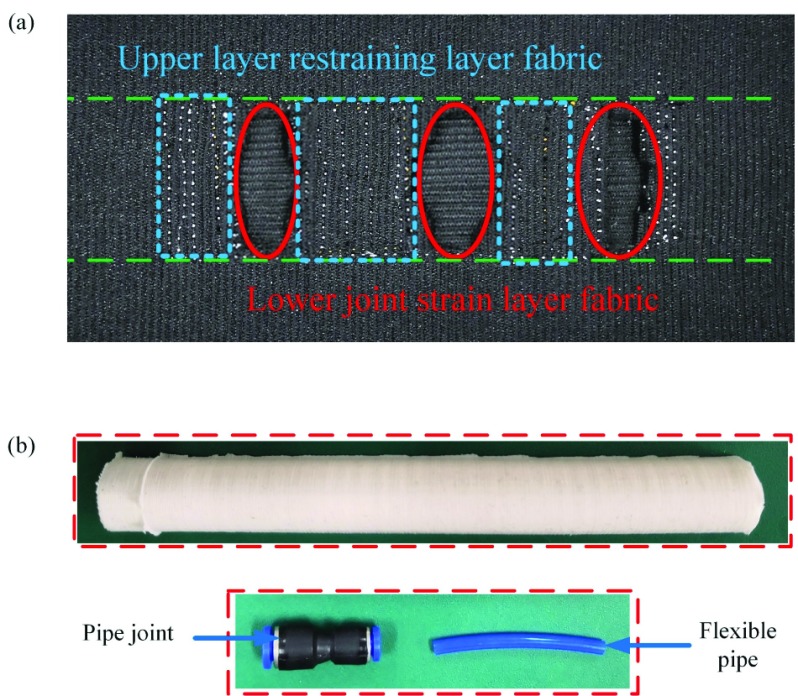
Fabrication process of fabric-based actuators. (a) Composite fabric for soft joint. (b) Soft actuator with auxiliary parts.

After the preparation of silica gel matrix, the fabric layer material is cut based on the design parameters of silica gel matrix, soft joint, phalanx and metacarpal. After the cutting is completed, the sewing machine is used to sew two layers of single-layer fabric along the joint edges (see Figure 5(a)). When the fabric layer composite cross-sewing is finished, the pipe joint is inserted into the hole and sealed with silica gel in the pressure inlet hole of silica gel matrix, and the flexible pipe is inserted into the pipe joint (see Figure 5(b)). Finally, the silica gel matrix is wrapped and stitched into the composite fabric layer. After the preparation of the five soft fingers, they are assembled and fixed together to form the whole rehabilitation glove.

## III. Experiment Verification of Soft Finger

To validate the proposed soft joint structure, experimental platform was constructed to study of the static characteristics of soft finger. The experimental platform is mainly composed of fluid-driven system, 3-DOFs test platform, 6-DOFs force sensor, pressure sensor, pressure transmitter and the signal acquisition system AI-01 module.

### A. Experimental Setup

**FIGURE 6.**
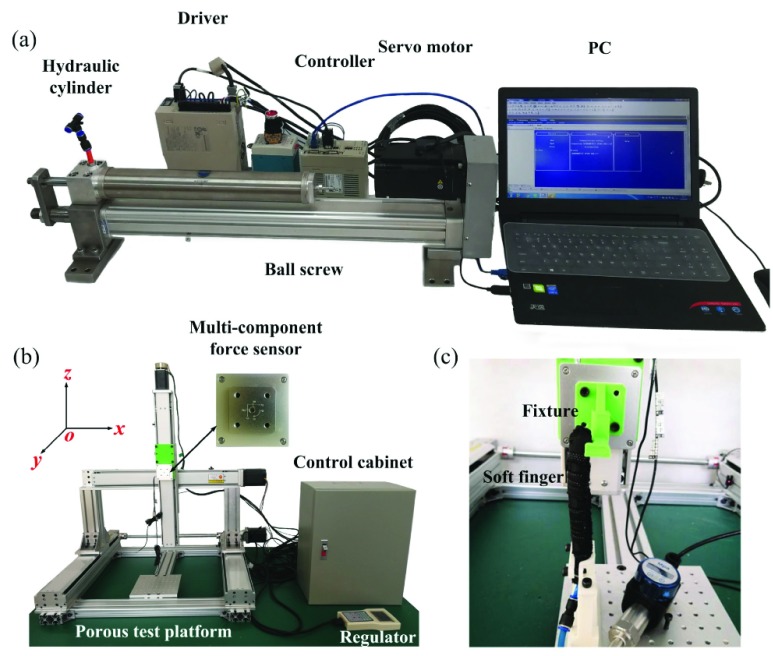
Configuration of test platform for the soft finger: (a) Fluid driven system. (b) 3DOFs test platform. (c) The fixture of finger joint test.

The proposed fluid-driven system, using servo motor hydraulic cylinder integrated structure, and the motor rotation is converted into the reciprocating linear feed motion of the ball screw slider through the supporting linear screw module between them. The reciprocating linear motion of the screw push rod drives the reciprocating action of the piston rod of the hydraulic cylinder to generate water pressure, and then directly drives the soft finger. The overall layout of the fluid-driven system given in Figure 6(a).

The 3-DOFs test platform is composed of three stepper motors, four sliding table modules, liquid crystal display control handle and a control cabinet (see Figure 6(b)). Through the 3-DOFs test platform, the coordinate of the multi-component force sensor space can be adjusted along with the terminal posture of the soft finger under different conditions. So that the multi-component force sensor can reach any spatial position within the measured range. Meanwhile, the LCD handle can record the moving distance of each axis in real time.

As shown in Figure 6(c), the soft finger was fixed on the base of the 3-DOFs test platform and the tip of the soft finger keep touch with the force sensor. In order to avoid the slip occurs between the finger and sensor, we made a soft finger fixture on the force sensor. For the test of each joint for a soft finger, we can adjust 3-DOFs test platform to make the fixture contact with each finger joint, and the output force of joints can be measured when the soft finger is pressurized.

### B. Characteristics of Soft Finger

By carrying out tests on the joint bending angle, trajectory and output torque of soft finger, we can have a more intuitive and in-depth understanding of the trajectory of soft finger as well as the internal law between the torque and fluid pressure, thus providing a theoretical basis for the development of rehabilitation glove.

The definition between the phalanx bone and joint angle of the soft rehabilitation finger is the same as that defined in the mechanical rehabilitation device. Each joint is taken as the joint point of the finger bone and the finger bone as the connecting rod, without considering the slight changes in the length and shape of the finger bone in the hand movement. Defined the rotation angle of each joint according to finger movement habit. According to the characteristics of human hand skeleton, when the hand is in motion, except for the thumb, the motion characteristics of the index finger, middle finger, ring finger and little finger are the same, and the movement actually occurs in only one plane. Taking index finger as an example, the joint bending experiment was studied.

#### 1) Angle of Soft Joints

The bending degree of the joints of soft fingers and the changing law of driving pressure can reflect the bending characteristics of the body to some extent, which directly affects the efficiency of rehabilitation gloves. In the research of joint motion angle, special points are used to mark the joints of the dorsal finger, so as to track and record the two-dimensional coordinates of each finger bone when the mollusk finger is compressed and bent with a 3-DOFs test platform, and then obtain the corresponding motion track of the three finger bones.

**FIGURE 7.**
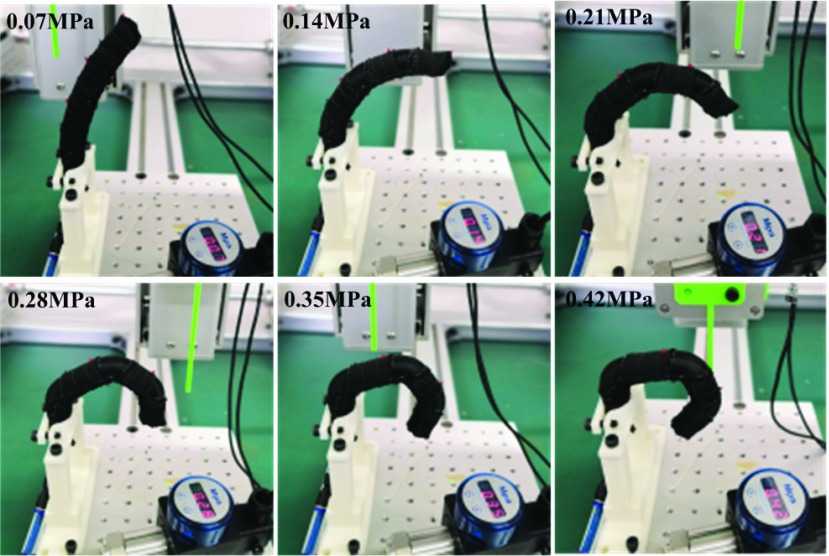
Bending test for soft finger.

In the experiment, the length of DP of the soft finger is 25 mm, the length of MP is 30mm, and the length of PP is 45 mm. Tests were carried out with the driving water pressure of 0.07MPa, 0.14MPa, 0.21MPa, 0.28MPa, 0.35MPa and 0.42MPa, respectively. During each test, the pointer installed on the 3-DOFs test platform was adjusted to coincide with the mark points on the back of the finger, and the 2d coordinates of the joints of the corresponding soft finger under each driving pressure were recorded, and the bending posture of the fingers was also captured (see Figure 7). It can be concluded that with the slow increase of driving pressure, the articular part of soft fingers is more distinct from the phalangeal part. Compared with the finger in the natural state, the fabric layer part designed at the phalanx maintains the original posture, while the interphalangeal joint presents obvious bending, which truly reflects the kinematic characteristics of human finger.

**FIGURE 8.**
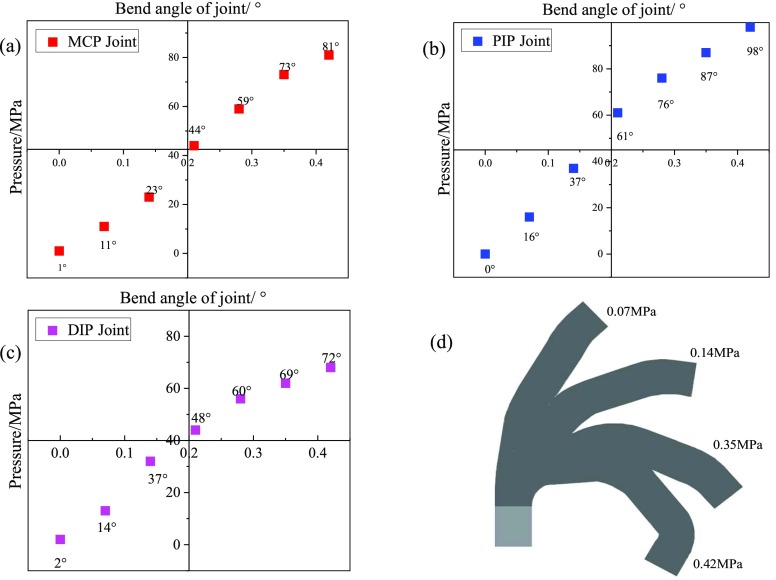
(a) Bending characteristics of MCP joint with pressure. (b) Bending characteristics of PIP joint with pressure. (c) Bending characteristics of DIP joint with pressure. (d) Sketch of a continuous curved contour of a finger.

The bending test results of the soft joints are given in Figure 8. Overall, all the three soft joint angle tend to increase with the increase of driving pressure. According to the slope of the test curves, the rate of angle change is relatively slow in the pressure range of 0 - 0.07MPa, compared with the pressure range of 0.07MPa - 0.14MPa. This is due to the physical properties of the silicone matrix inflating at low pressure and the composite elastic fabric layer having slight deformation as well as the sealing stitching line fiber with low elastic, so the soft fingers will expand radially before bending.

Therefore, under the initial state of low pressure, soft finger is not particularly sensitive to the bending characteristics of pressure. In the pressure range of 0.14MPa - 0.42MPa, the response rate of the three joints of soft finger to pressure shows a trend of gradually increasing first and then gradually decreasing. Because soft finger movement is weakened by radial expansion, each joint responds quickly with pressure changes. As the pressure increases, the bending angle of each joint increases, and the elongation of the fabric layer is close to the limit value, so the rate of angle increase slows down. When driving pressure is 0.42MPa, the bending angles of the three joints reached 81°, 98° and 72° respectively, shows that the soft finger structure design is reasonable and has good effect of bending. In addition, due to the large bending deformation of the silicon matrix itself, the middle part will produce greater resilience compared with other parts. Therefore, the minimum bending angle of DIP joint is 72° ultimately.

#### 2) Output Torque of Soft Joints

**FIGURE 9.**
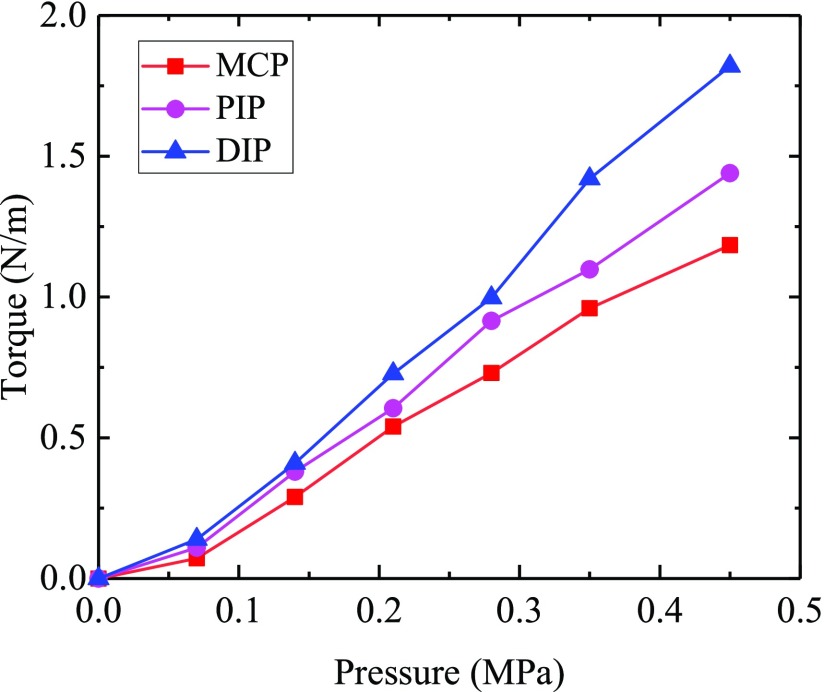
Output force of the tip of soft finger with driving pressure.

The output torque of joints and its variation rule determine the maximum pressure that soft finger can bear and directly affect the hand rehabilitation effect. Depending on the patient’s condition, the output torque can be increased under greater pressure. Output torque measuring curve of the soft finger shows that the output torque of each joint of the soft finger increases gradually with the same amount of driving pressure (see Figure 9).

When the driving pressure is 0.42MPa, MCP joint, PIP joint and DIP joint can produce output torque of 1.18Nm, 1.44Nm and 1.82Nm respectively. Furthermore, the MCP joint produces the smallest driving torque and the DIP joint the largest, as the pressure increases, the torque change rate of DIP joint is faster than that of MCP joint and PIP joint, and the torque change rate increases first and then decreases. This is due to the special structural design of the soft finger joint, which makes the interphalangeal joint becomes a small constant curvature arc when the soft finger is bent, which can be regarded as a continuous soft element. At first, due to expansion, the output torque is small. When the pressure keeps increasing, the output torque keeps increasing, due to the soft finger ontology deformation is larger, the deformation recovery force generated is larger, which has a significant impact on the output torque, so the increase trend of output torque becomes slow.

## IV. Rehabilitation Training Test

### A. Performance Evaluation

To verify the performance of the soft rehabilitation glove system, control system based on surface electromyography (sEMG) was constructed and continuous passive training (CPM) test was carried out for the whole system.

#### 1) Control System

**FIGURE 10.**
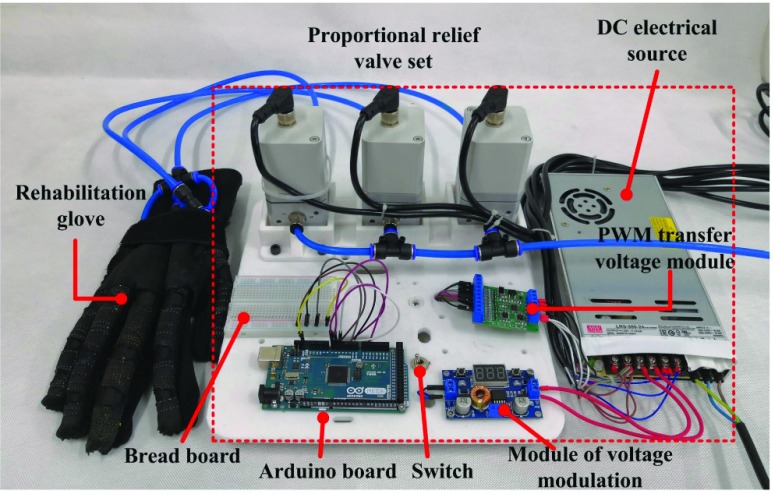
Control system of the soft rehabilitation glove.

This paper uses compressed air as power source to drive and control soft rehabilitation glove. The pneumatic control system is mainly composed of sEMG acquisition system, Arduino controller, proportional regulating valve of FESTO, PWM voltage conversion module and power supply module. The physical picture of the rehabilitation glove control system can be seen in Figure 10. Its basic functions are as follows: write programs in the Arduino IDE software and download the programs into the Arduino controller to run the programs. The sEMG acquisition system sends the action instruction to the controller through the serial port, controller receives the action instructions and generates the corresponding duty cycle PWM wave according to the control signal, and the digital output of PWM is converted into the corresponding analog voltage signal through the PWM voltage module. The proportional reducing valve adjusts the driving pressure of the soft glove according to the analog voltage signal. Above them, the acquisition system can collect the patient’s hand sEMG of the forearm, and for patients with residual muscle strength, residual EMGs of the affected hand can be collected, which can be used to passive and active control of the affected hand. It mainly includes two modules: the training process of gesture and the recognition output. The subjects need to carry out the preset motion training and save it, and then repeat the preset motion as the control input to control the soft finger movement.

#### 2) Continuous Passive Training Test

**FIGURE 11.**
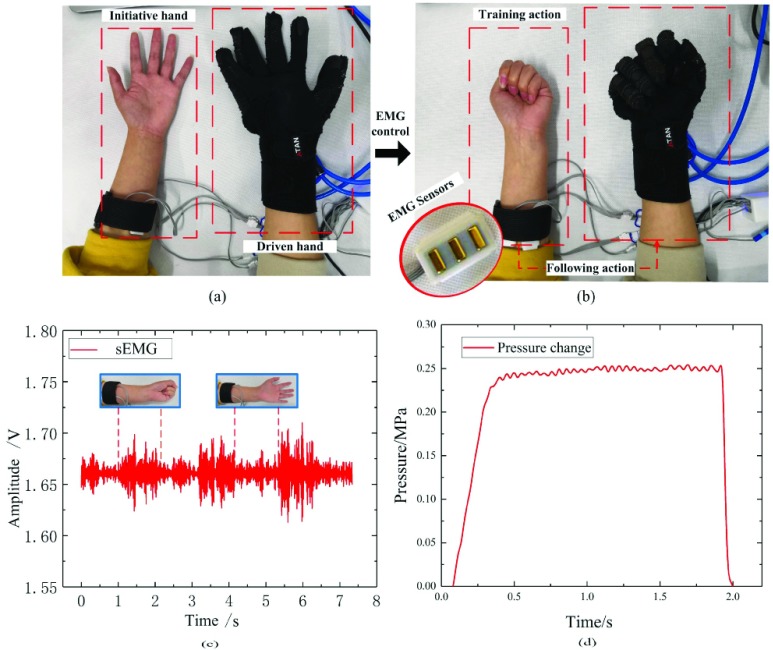
(a) Passive training of hand stretching. (b) Passive training of hand stretching. (c) Continuous passive training EMG signal. (d) Pressure curve of periodic passive training.

For the later treatment of patients with hand dysfunction caused by spinal cord injury or cerebral apoplexy, rehabilitation therapy is mainly adopted. Above them, the most important exercise rehabilitation therapy is CPM. During the experiment, the electrodes of the sEMG sensor were put on the forearm of the subject, the soft rehabilitation glove was put on the other hand of the subject. The subjects slowly and egularly perform repetitive motions of hand stretching (see Figure 11(a)) and contracting at a certain rate (see Figure 11(b)). The control system of soft rehabilitation glove collect the sEMG of the arm where the subject wears the electrode and control the glove to drive the other hand of the subject to generate the “mirror” follow action. At the same time, the subject can clearly feel the squeezing force of the glove driving actuators on each finger, which makes each finger generate passive movement.

A measured curve of sEMG for three periods of continuous passive training are given in Figure 11(c), these results can reflect the change of sEMG signal intensity when the fingers are stretching and contracting. These signals will be collected into the control system of soft gloves and judged to be the specific control actions of rehabilitation. The corresponding $x$-coordinate periods are 0~1 s, 2.2s~3.2 s, 4.3 s~5.4 s, 6.6 s~7.4 s when the finger joints are stretched, and the sEMG amplitude is about 0.016v. The rest of the time is the state of finger contraction, and the sEMG amplitude is larger about 0.04v. sEMG detected can clearly reflect the movement state of the associated muscles during finger joint movement. In addition, the measured curve of sEMG signal detected shows regular and stable periodic changes, which can clearly reflect the motion state of the associated muscles during finger movement.

Considering the grasping characteristics of human hands and the resistance of patients’ hands, it is set that the pressure will increase slowly when the fingers contract, and rapidly relieve pressure when the fingers are stretched, and the highest pressure can be adjusted for patients under different conditions. According to the requirements of hand rehabilitation, the driving pressure range is set 0~0.25MPa in this experiment, the period of passive training of this subject is about 2s, the cycle of passive training is about 30 times /min. The pressure arise time is 0.32s, the stability interval is 0.613s and the pressure drop time is about 0.067s. Pressure changes in the rehabilitation gloves during continuous passive extension and convergence exercise training for one period is shown in Figure 11(d).

#### 3) Grasping Performance Test

**FIGURE 12.**
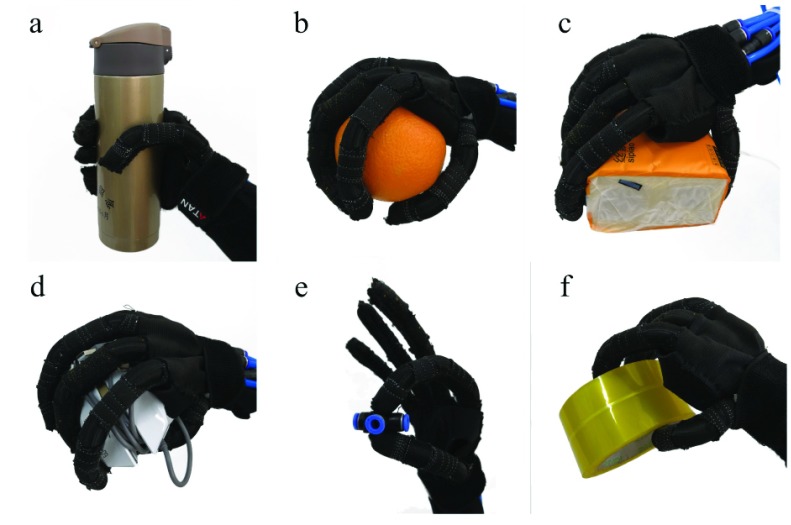
The grasping test for different shapes of objects. (a) Thermos cup. (b) Orange. (c) Tissue box. (d) Mouse. (e) Pneumatic pipe joint. (f) Tape roll.

In order to verify the grasping flexibility of this fabric reinforced soft rehabilitation glove, the glove itself (not worn on the hand) was tested for the grasping of objects with different shapes as shown in Figure 12. As we can see from the test results of object grasping with different appearances, materials and volumes, we can conclude that the soft rehabilitation gloves designed in this paper can take the initiative to deform according to the shape of the external contour of the object to be grasped. It has flexible grasping ability for different shapes and small objects, which is suitable for assisting patients with muscular disorders in everyday life.

## V. Conclusion

In this work, a fabric-reinforced soft rehabilitation glove is proposed, and the soft joint structure is discussed by referring to the movement mechanism of human fingers, based on composite elastic fabric. The fabric layer is selected from commonly used textile industry fabrics, the tensile properties are experimentally analyzed, and the optimum design of the soft joint is carried out through theoretical analysis. The fabric is transformed from a single layer structure into a multi-layer composite structure with specific physical characteristics. To verify the proposed soft rehabilitation glove including specific functions, bending angle, motion trajectory, and output torque, experiments are carried for the soft finger and glove, and the relevant conclusions are obtained as follows:

1) Warp and weft tensile tests are carried out on the tensile testing machine for the composite warp-knitted fabric. Experimental results report that the maximum tension is 22N, the warp deformation is 1.76mm, the elongation is 2.7%, and the weft deformation is 65mm, the elongation is 100%.
2) When the fluid pressure is 0.42 MPa, the bending angle of soft joints of MCP, PIP and DIP is up to 81°, 98°, 72° and can produce output torque of 1.18Nm, 1.44Nm, 1.82Nm respectively, and the joint trajectory of soft fingers conforms to the characteristics of human hands, which demonstrates that the structure design of soft finger is reasonable.
3) The results show that the fabric-reinforced joint of soft rehabilitation glove can adapt to the external contour shape of the captured object. The soft rehabilitation glove controlled by EMG system can complete continuous training and have the function of assisting daily life articles to grasp.
4) Although our work has made good progress in structural design and preliminary test of the soft rehabilitation glove, there are still a lot of works to be done, including optimization of the glove control system, further active treatment methods and more clinical tests for PD patients. Next step, we will carry out more in-depth research in these areas.
